# Relating soil physical properties to other soil properties and crop yields

**DOI:** 10.1038/s41598-022-26619-8

**Published:** 2022-12-20

**Authors:** Upendra M. Sainju, Daniel Liptzin, Jalal D. Jabro

**Affiliations:** 1 USDA-ARS, Northern Plains Agricultural Research Service, Sidney, MT 59270 USA; 2 Soil Health Institute, Morrisville, NC 27560 USA

**Keywords:** Plant sciences, Biogeochemistry

## Abstract

Soil physical properties can be related to other soil properties and crop yields, but their evaluations as soil health indicators relating to extensive soil properties and long-term crop yields need further exploration. We evaluated the long-term (14 and 36 year) effect of cropping systems and N fertilizations on selected soil physical properties and related them to 66 soil physical, chemical, biological, and biochemical properties and mean crop yields across years at two dryland farming sites in the semiarid region of the northern Great Plains, USA. Treatments were rotations of no-tillage and conventional tillage spring wheat (*Triticum aestivum* L.), barley (*Hordeum vulgare* L.), pea (*Pisum sativum* L.), and fallow with or without N fertilization. Soil samples collected in April 2019 were analyzed for physical, chemical, biological, and biochemical properties and mean crop yields were determined. The average slake aggregate (ASA), wet aggregate stability index (WASI), and intact core available water holding capacity (IAWHC) were associated with most soil physical, chemical, biological, and biochemical properties and clay concentration was associated with nutrient concentrations. These parameters were also better related to mean crop yields across years than other soil physical properties. Because of the enhanced relationship with soil properties and crop yields and simple and inexpensive measurement, ASA can be used as a potential soil health indicator in dryland cropping systems in semiarid regions.

## Introduction

Soil physical properties as important components of soil health influence water and nutrient movements, aeration, soil temperature, nutrient cycling, and root growth that affect crop yields and environmental quality^1–3^. For example, increased bulk density (BD) due to increased soil compaction results in decreased pore volume that reduces water infiltration, increases aeration stress, lowers soil temperature and nutrient cycling, increases denitrification, losses mycorrhizal fungi, and reduces root growth^2^. In contrast, increased soil aggregation enhances water and nutrient movements, reduces soil erosion, promotes C sequestration, favors microbial activity and abundance, and increases root growth and crop yields^1,3^. Clay concentration is an important indicator of soil health that enhances the retention of soil water and nutrients^4^. While increased soil water retention enhances crop yields^2,5^, reduced water infiltration capacity of the soil results in anaerobic condition that hampers nutrient cycling and root growth, thereby reducing crop production^1,5^.

Some soil physical properties are related to other soil properties^4,6,8,11,12^. Numerous researchers^1,6^^,^^7^ reported that BD was negatively correlated to soil organic C (SOC), but soil aggregation, water retention, and water infiltration capacity were positively correlated to SOC. In contrast, Nemes et al.^8^ observed that soil hydraulic conductivity (SHC) was negatively correlated to SOC because soils rich in organic matter have lower water permeability. Blanco-Canqui and Benjamin^1^ concluded that the relationship between soil physical properties and SOC are affected by soil and climatic conditions and management practices. Shaver et al.^9^ found that soil aggregation was related to clay concentration. Norkaew et al.^4^ showed that water-stable aggregation (WSA) was positively related to SOC, soil total N (STN), potential N mineralization (PNM), and microbially active C (MAC), but BD was negatively related to these parameters. Shestak and Busse^10^ found that BD was negatively correlated to macroporosity (MAP), but was not related to microbial properties. Stott et al.^11^ reported that soil aggregation and water infiltration capacity were positively correlated, but BD was negatively correlated to enzyme activities, such as β-glucosidase (BG). Similarly, several researchers^12,13^ demonstrated that BD was positively correlated, but SHC was negatively correlated to activities of N-acetyl-β-glucosamidinase (NAG) and phosphomonoesterase (PME).

Soil physical properties also vary in their relationships to crop production. Sene et al.^14^ found that sand concentration and SHC were positively correlated, but soil aggregation was negatively correlated to crop yield. Shang et al.^15^ reported that soil water content and aggregate stability (AS) were positively related, but BD was negatively related to crop yield. Similarly, several researchers^16,17^ observed that BD was negatively related to crop yield. In contrast, Logsdon and Karlen^2^ showed that BD was not related to crop yield. Maddoni et al.^3^ showed that AS was an important indicator of soil health that related better to corn (*Zea mays* L.) yield than labile C and N fractions.

Although some soil physical properties are related to other soil properties and crop yields, an in-depth examination of their relationships with other soil properties and long-term crop yields are needed for their proper evaluation for potential soil health indicators. We chose six soil physical properties (clay concentration, BD, average slake aggregate [ASA], wet aggregate stability index [WASI], intact core available water capacity (IAWHC], and SHC) representing particle size, soil aggregation, compaction, water retention, and water infiltration capacity that have a broad impact on soil health, such as water and nutrient movements, aeration, soil temperature, C sequestration, nutrient cycling, and microbial properties and related them to 66 other soil properties and long-term crop yields at two dryland farming sites in eastern Montana, USA. We hypothesized that these physical properties would relate to other soil properties and long-term crop yields and that some physical properties may be used as potential soil health indicators due to their close relationships with the above parameters in dryland cropping systems in the semiarid region. Our objectives were to: (1) determine if physical properties are related to other 66 other soil physical, chemical, biological, and biochemical properties, and (2) select which soil physical properties can be used as potential soil health indicators that are related to most soil properties and crop yields.

## Results

### Relationships among soil properties

#### Soil physical properties

At Froid, clay concentration was positively correlated to WASI (Table 1). The ASA was positively correlated to WASI and IAWHC, but negatively correlated to BD. The WASI was positively correlated to IAWHC and SHC. The BD was negatively correlated to IAWHC. At Sidney, WASI was positively correlated to ASA and IAWHC. Similarly, SHC was positively correlated to IAWHC.

**Table 1:** Correlation (r) among clay concentration (Clay), average slake aggregate (ASA), wet aggregate stability index (WASI), bulk density (BD), intact core available water holding capacity (IAWHC), and saturated hydraulic conductivity (SHC) at Froid (n = 16) and Sidney (n = 24), Montana.

Soil physical properties	Clay	ASA	WASI	BD	IAWHC	SHC
**Froid**						
Clay (g kg^−1^)	–	0.14	0.50*	− 0.32	0.36	0.14
ASA	0.14	–	0.66**	− 0.54*	0.52*	0.28
WASI	0.50*	0.66**	–	− 0.38	0.51*	0.69**
BD (Mg m^−3^)	− 0.32	− 0.54*	− 0.38	–	− 0.71**	− 0.13
IAWHC (cm^3^ cm^−3^)	0.36	0.52*	0.51*	− 0.71**	–	0.14
SHC (cm h^−1^)	0.14	0.28	0.69**	− 0.13	0.14	–
**Sidney**						
Clay (g kg^−1^)	–	− 0.25	− 0.24	0.05	0.34	0.17
ASA	− 0.25	–	0.67**	− 0.22	0.38	0.11
WASI	− 0.24	0.67**	–	− 0.36	0.46*	0.32
BD (Mg m^−3^)	0.05	− 0.22	− 0.36	–	0.08	0.15
IAWHC (cm^3^ cm^−3^)	0.34	0.38	0.46*	0.08	–	0.43*
SHC (cm h^−1^)	0.17	0.11	0.32	0.15	0.43*	–

* and ** Significant at *P* ≤ 0.05 and 0.01, respectively.

**Figure 1:**
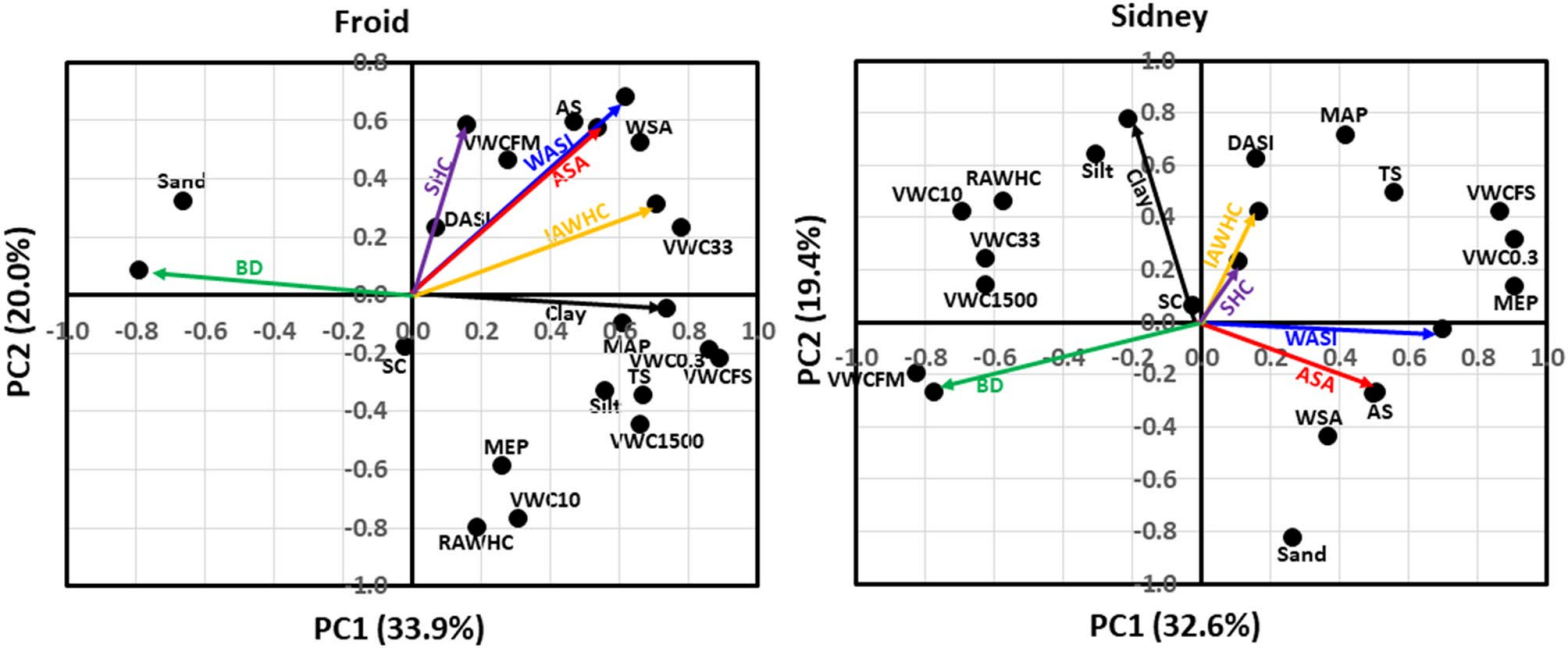
Principal component analysis for the associations among average slake aggregate (ASA), bulk density (BD), clay concentration, intact core available water capacity (IAWHC), saturated hydraulic conductivity (SHC), wet aggregate stability index (WASI), and soil physical properties at Froid and Sidney, MT. At Froid, soil physical properties explained 33.9% of the total variability in the first principal component (PC1) and 20.0% in the second principal component (PC2). At Sidney, physical properties explained 32.6% of total variability in PC1 and 19.4% of the total variability in PC2. AS denotes aggregate stability; DASI, dry aggregate stability index; MAP, macro-porosity; MEP, meso-porosity; RAWHC, repacked core available water capacity; SC, stone content; TS, total shrinkage; VWC0.3, volumetric water content at 0.3 kPa; VWC10, volumetric water content at 10 kPa; VWC33, volumetric water content at 33 kPa; VWC1500, volumetric water content at 1500 kPa; VWCFM, volumetric water content in the field-moist soil; VWCFS; volumetric water content at water saturation; and WSA, water-stable aggregation.

The principal component analysis (PCA) indicated that soil physical properties contributed 34% of the total variability in principal component 1 (PC1) and 20% variability in principal component 2 (PC2) at Froid (Fig. 1). Clay concentration was positively associated with volumetric water content at 0.3 and 33 kPa (VWC0.3 and VWC33, respectively), volumetric water content at water saturation (VWCFS), and MAP, but negatively associated with BD. The IAWHC was positively associated with VWC33 and WSA. The ASA and WASI were positively associated with WSA, AS, and volumetric water content at the field-moist soil (VWCFM). The SHC was positively associated with VWCFM and dry aggregate stability index (DASI), but negatively associated with stone content (SC). Soil physical properties that were not associated with clay concentration, ASA, WASI, IAWHC, SHC, and BD were sand concentration, repacked core available water holding capacity (RAWHC), volumetric water content at 10 kPa (VWC10), mesoporosity (MEP), volumetric water content at 1500 kPa (VWC1500), silt concentration, and total shrinkage (TS).

At Sidney, soil physical properties contributed 33% of the total variability in PC1 and 19% of the variability in PC2 (Fig. 1). The ASA was positively associated with AS and WSA and negatively associated with VWC33, VWC10, RAWHC, and VWC1500. The WASI was positively associated with MEP, VWC0.3, and AS, but negatively associated with VWC1500. The IAWHC was positively associated with DASI. The BD was positively associated with VWCFM, but negatively associated with VWC0.3. The clay concentration was positively associated with silt concentration, but negatively associated with sand concentration. Soil physical properties that were not associated with clay concentration, ASA, WASI, IAWHC, SHC, and BD were VWCFS, TS, MAP, RAWHC, and SC.

#### Soil chemical properties

**Figure 2:**
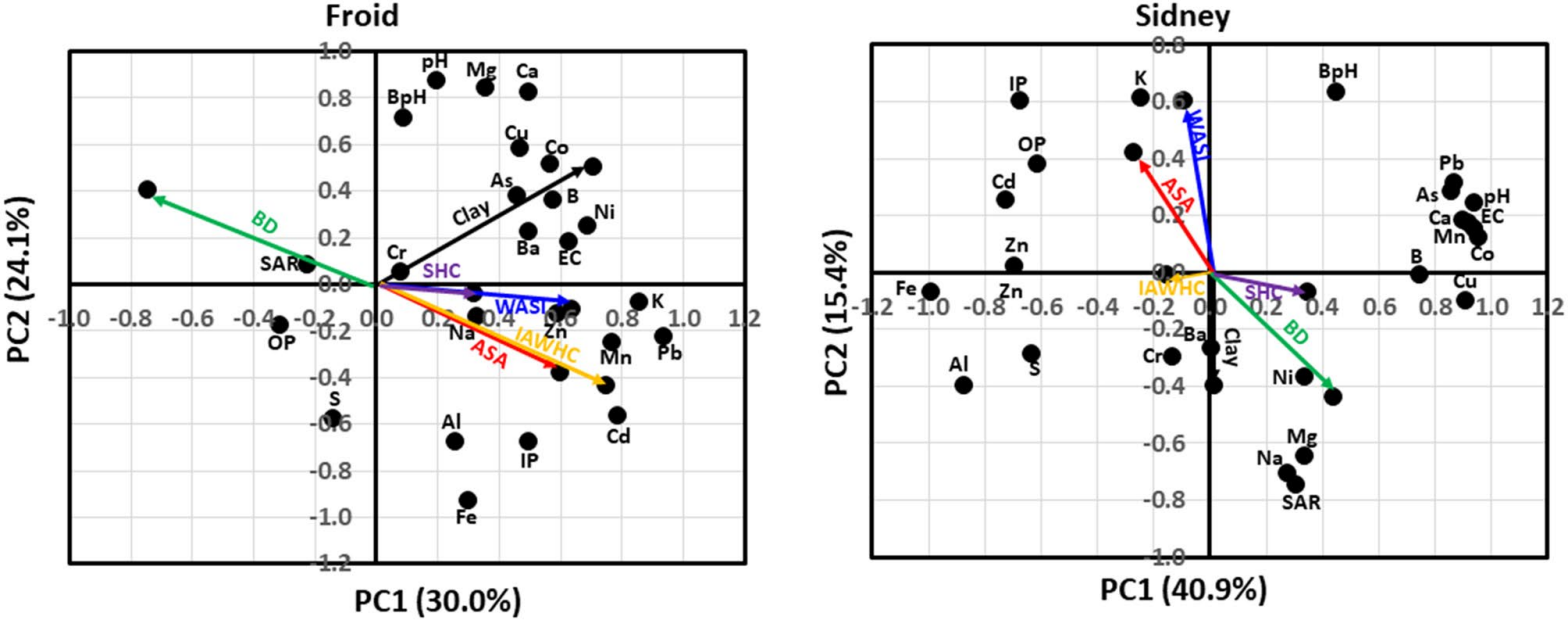
Principal component analysis for the associations among average slake aggregate (ASA), bulk density (BD), clay concentration, intact core available water capacity (IAWHC), saturated hydraulic conductivity (SHC), wet aggregate stability index (WASI), and soil chemical properties at Froid and Sidney, MT. At Froid, soil chemical properties explained 30.0% of the total variability in the first principal component (PC1) and 24.1% in the second principal component (PC2). At Sidney, chemical properties explained 40.9% of total variability in PC1 and 15.4% in PC2. BpH denotes buffer pH; EC, electrical conductivity; IP, inorganic P concentration; OP, organic P concentration; and SAR, sodium-absorption ratio.

In the PCA, soil chemical properties contributed 30% of the total variability in PC1 and 24% of the variability in PC2 at Froid (Fig. 2). The ASA and IAWHC were positively associated with Cd, inorganic P (IP), Mn, Pb, K, and Zn concentrations, but negatively associated with Na-absorption ratio (SAR). The WASI was positively associated with electrical conductivity (EC) and Zn, Mn, Pb, and K concentrations. The SHC was positively associated with Na concentration. Clay concentration was positively associated with EC and Cu, Co, B, As, Ba, and Ni concentrations, but negatively associated with organic P (OP) concentration. The BD was positively associated with SAR, but negatively associated with Cd concentration. Soil chemical properties that were not associated with ASA, BD, Clay, IAWHC, SHC, and WASI were pH, buffer pH (BpH), and Al, Fe, Ca, Mg, and S concentrations.

At Sidney, chemical properties contributed 41% of the total variability in PC1 and 15% of the variability in PC2 (Fig. 2). The ASA was positively associated with K, IP, and OP concentrations, but negatively associated with Ni and Mg concentrations. The WASI was positively associated with K concentration, but negatively associated with Ba concentration. The BD was positively associated with Ni and Mg concentrations. Clay concentration was positively associated with Ba and Cr concentrations. Soil chemical properties that were not associated with ASA, BD, Clay, IAWHC, SHC, and WASI were pH, BpH, EC, SAR, and Pb, As, Ca, Mg, Co, B, Cu, Na, Al, MN, S, Fe, Zn, Cd, OP, and IP concentrations.

#### Soil biological and biochemical properties

**Figure 3:**
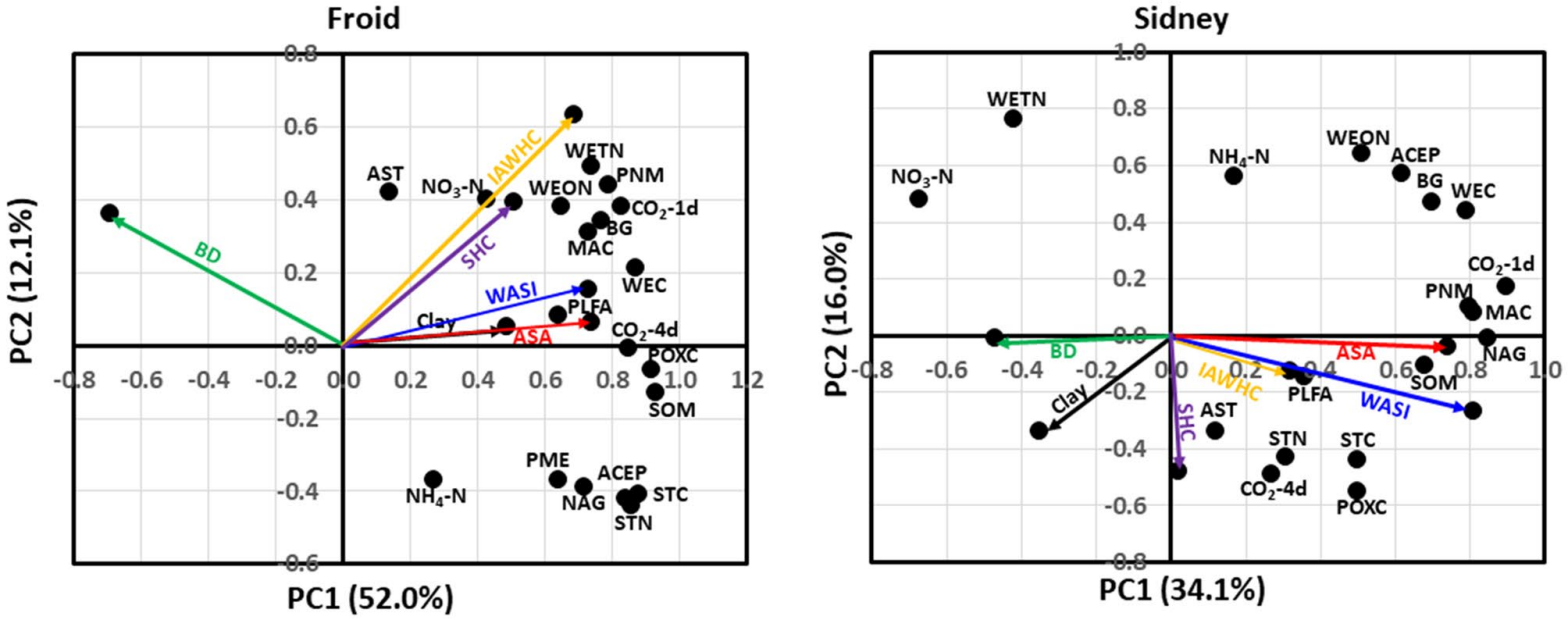
Principal component analysis for the associations among average slake aggregate (ASA), bulk density (BD), clay concentration, intact core available water capacity (IAWHC), saturated hydraulic conductivity (SHC), wet aggregate stability index (WASI), and soil biological and biochemical properties at Froid and Sidney, MT. At Froid, soil biological and biochemical properties explained 52.0% of the total variability in the first principal component (PC1) and 12.1% in the second principal component (PC2). At Sidney, biological and biochemical properties explained 34.1% of the total variability in PC1 and 16.9% in PC2. ACEP denotes autoclaved citrate-extractable protein; AST, arylsulfatase; BG, β-glucosidase; CO_2_-1d, CO_2_ flush at 1 d incubation; CO_2_-4d, CO_2_ flush at 4 d incubation; MAC, microbially active C; NAG, N-acetyl β-glucosaminidase; PLFA, phospholipid-derived fatty acid; PME, phosphomonoesterase; PNM, potentially N mineralization; POXC, KMnO_4_-extractable C; SOM, soil organic matter; STC, soil total C; STN, soil total N; WEC, water-extractable C; WEON, water-extractable N; and WETN, water-extractable total N.

At Froid, the PCA indicated that soil biological and biochemical properties contributed 52% of the total variability in PC1 and 12% of the variability in PC2 (Fig. 3). The ASA and WASI were positively associated with phospholipid-derived fatty acid (PLFA), water-extractable C (WEC), CO_2_ flush at 4-d incubation (CO_2_-4d), KMnO_4_-extractable C (POXC), soil organic matter (SOM), MAC, BG, and CO_2_ flush at 1-d incubation (CO_2_-1d). The IAWHC was positively associated with water-extractable total N (WETN), PNM, water-extractable organic N (WEON), CO_2_-1d, and NO_3_-N. Clay concentration was positively associated with PLFA. The SHC was positively associated with NO_3_-N and WEON. In contrast, BD was negatively associated with PME, autoclaved citrate-extractable protein (ACEP), NAG, soil total C (STC), and STN. Soil biological and biochemical properties that were not associated with ASA, BD, Clay, IAWHC, SHC, and WASI were arylsulfatase (AST), NH_4_-N, PME, NAG, STC, and STN.

At Sidney, soil biological and biochemical properties contributed 34% of the total variability in PC1 and 16% of the variability in PC2 (Fig. 3). The ASA was positively associated with PNM, MAC, CO_2_-1d, NAG, and SOM. The WASI was positively associated with SOM, NAG, and STC. The IAWHC was positively associated with PLFA. The SHC was positively associated with AST. In contrast, clay concentration was negatively associated with ACEP, WEON, and BG. The BD was negatively associated with NAG, SOM, and MAC. Soil biological and biochemical properties that were not associated with ASA, BD, Clay, IAWHC, SHC, and WASI were NO_3_-N, WETN, NH_4_-N, WEC, STN, CO_2_-4d, and POXC.

#### Relationship between soil physical properties and crop yields

**Figure 4:**
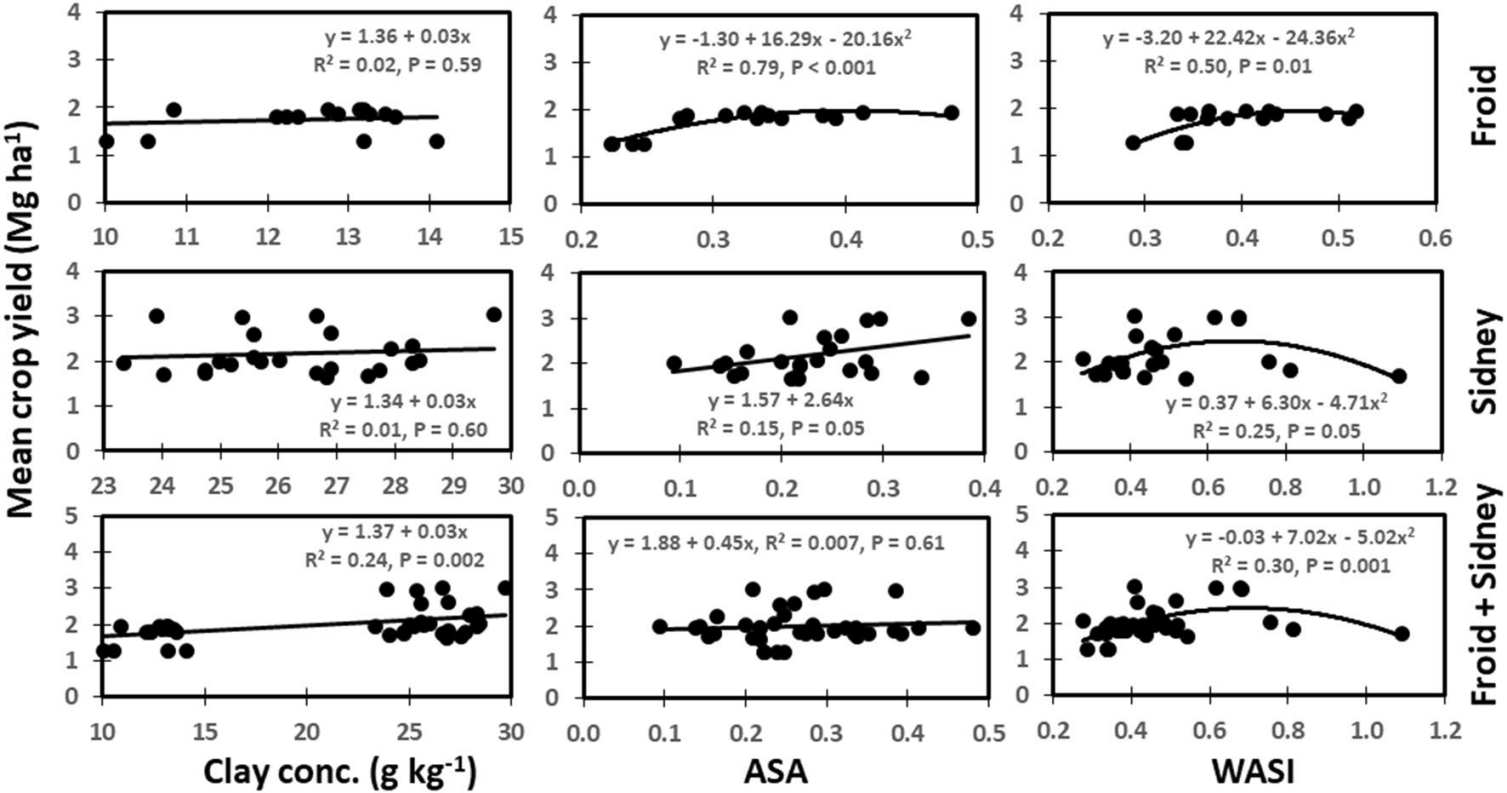
Relationships among clay concentration, average slake aggregate (ASA), wet aggregate stability index (WASI), and mean crop yield across years at Froid, Sidney, and the combined data from Froid and Sidney.

Clay concentration was strongly (*P* ≤ 0.01) related to mean crop yield across years from the combined data from Froid and Sidney, but not for the individual sites (Fig. 4). Clay concentration accounted for 24% of the variability in crop yield. An increase in clay concentration by 1 g kg^−1^ increased crop yield by 0.03 Mg ha^−1^. The ASA was very strongly (*P* ≤ 0.001) related to mean crop yield at Froid and weakly (*P* ≤ 0.05) related at Sidney. The ASA accounted for 79% of the variability in crop yield at Froid and 15% of the variability at Sidney. The WASI was strongly related to mean crop yield at Froid, weakly related at Sidney, and very strongly related to the combined data from Froid and Sidney. The WASI accounted for 50% of the variability in crop yield at Froid, 25% of the variability at Sidney, and 30% of the variability for the combined data from Froid and Sidney.

**Figure 5:**
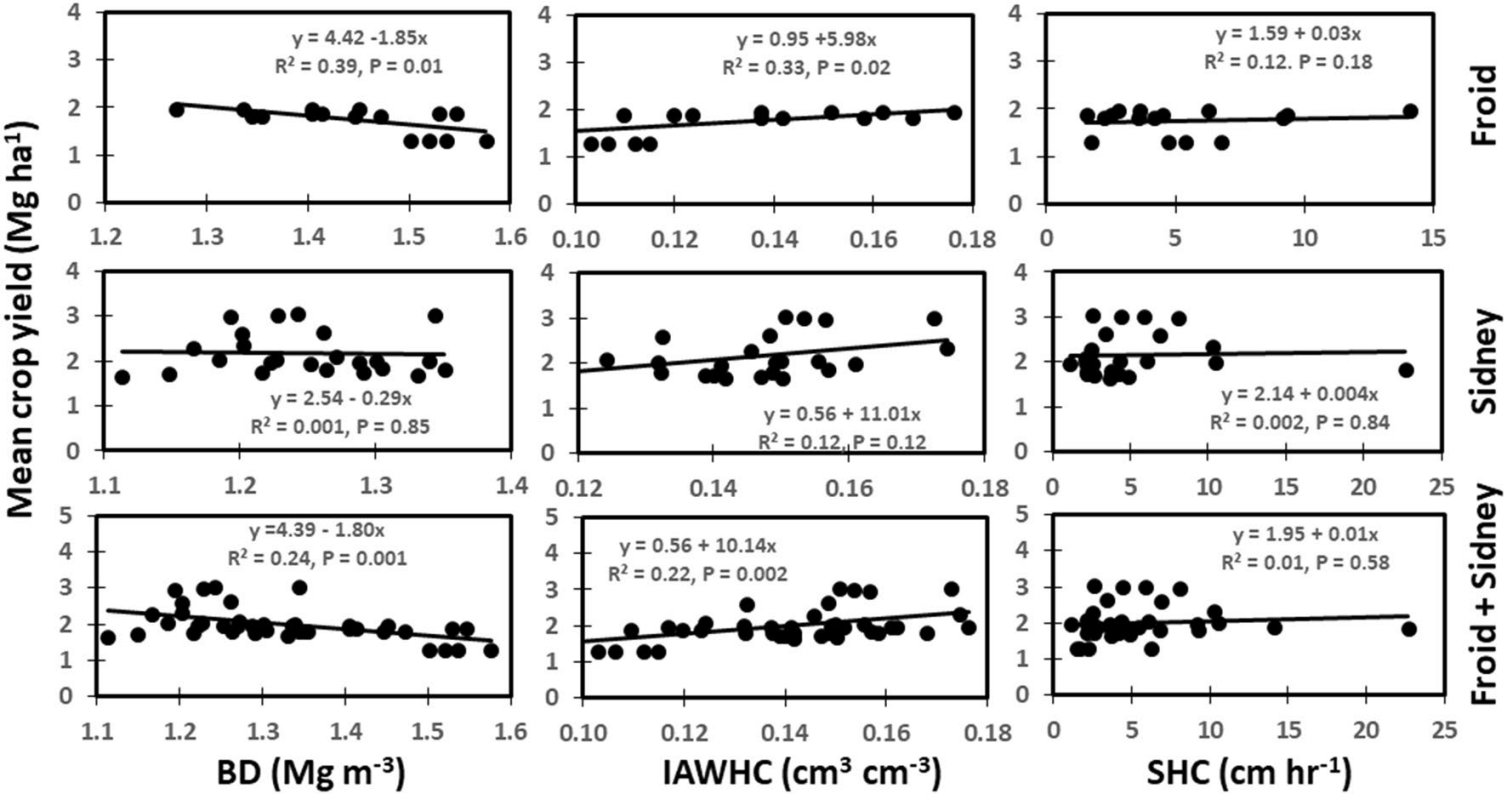
Relationships among soil bulk density (BD), intact core available water capacity (IAWHC), saturated hydraulic conductivity (SHC), and mean crop yield across years at Froid, Sidney, and the combined data from Froid and Sidney.

The BD was the only soil physical property that negatively related to mean crop yield (Fig. 5). The BD was strongly related to crop yield at Froid and very strongly related to the combined data from Froid and Sidney (Fig. 5). The BD accounted for 39% of the variability in crop yield at Froid and 24% variability for the combined data from Froid and Sidney. An increase in BD by 1 Mg m^−3^ decreased crop yield by 1.85 Mg ha^−1^ at Froid and 1.80 Mg ha^−1^ for the combined data from Froid and Sidney. The IAWHC was weakly related to mean crop yield at Froid and strongly related to the combined data from Froid and Sidney. The IAWHC accounted for 33% of the variability in crop yield at Froid and 22% variability for the combined data from Froid and Sidney. The SHC was not related to crop yields at both sites or to the combined data from Froid and Sidney.

## Discussion

### Relationship among soil properties

The positive correlations among ASA, WASI, IAWHC, and SHC at Froid and Sidney suggest that well aggregated soils can also enhance soil water retention and infiltration capacities. As soil aggregation is related to SOC^1^, it is possible that increased SOC in enhanced aggregated soil helped to retain more water. Also increased soil aggregation may have increased macroporosity which increased SHC. In contrast, negative correlations among BD, ASA, and IAWHC at Froid suggest that increased soil compaction can reduce slaking resistance of soil aggregates and water holding capacity. Blanco-Canqui and Benjamin^1^ reported that well aggregated soils have lower BD, better consistency, and greater macropores and water retention capacity than poorly aggregated soils. Benjamin et al.^18^ found that soil aggregation was positively correlated to SHC, but negatively correlated to BD. Manrique and Jones^19^ reported that BD was negatively correlated to soil water content. Our result of positive correlation between clay concentration and WASI was similar to that observed by Shaver et al.^9^.

The positive associations among ASA, WASI, WSA, AS, VWC33, IAWHC, and VWCFM at Froid and Sidney suggest that increased soil aggregation enhanced aggregate stability, slaking resistance, and water holding capacity. Water-stable aggregates with enhanced stability have higher slaking resistance and water holding capacity and are less erodible than aggregates with reduced stability^1^. The close association between IAWHC and VWC33 at Froid indicates that water holding capacity increased as soil water content increased. Similarly, the close association between SHC and VWCFM suggests that the water infiltration rate increased as the soil water content at saturation increased. The negative association between clay concentration and BD suggests that soils with high clay concentration are less dense than soils with less concentration. Similarly, the negative association between clay and sand concentrations at both sites suggests that clay concentration increased as sand concentration decreased.

The positive associations among ASA, WASI, and some nutrients at Froid and Sidney suggest that improved soil aggregation can favor retention and availability of nutrients probably by enhancing nutrient cycling. As soil aggregation is closely related to SOC^1,6^, it is likely that increased SOC that enhanced soil aggregation also favored nutrient cycling. Similarly, the positive associations among IAWHC and some nutrients indicate that increased water holding capacity of soil also may help to retain nutrients that may be leached out of the soil profile. Increased clay concentration also may have favored the retention of nutrients, as clay concentration was positively associated with nutrients at both sites. Negatively charged clay particles may have attracted cations, helping to retain positively charged nutrients. The BD and SHC had minor associations with soil chemical properties, as soil compaction and water infiltration capacity may be less important for affecting chemical properties and nutrient levels.

A greater number of soil chemical properties were associated with ASA, BD, Clay, IAWHC, SHC, and WASI at Froid than at Sidney (Fig. 2). This suggests that the longer duration of the experiment conducted in coarse-textured soil may have altered both soil physical and chemical properties that were more related to each other than the shorter duration of the experiment conducted in fine-textured soil. This indicates that long duration experiments may be more ideal to evaluate soil physical properties as potential soil health indicators relating to chemical properties than short duration experiments in dryland cropping systems.

The positive associations among ASA, WASI, and most biological and biochemical properties reveal that soil aggregation is related to SOM, microbial abundance and activity, nitrogen mineralization, and enzyme activities. The binding agents from soil organic matter and fungal growth enhance soil aggregation^1^, as fungi contribute more to soil aggregation than bacteria because of their greater abundance^20,21^. Some researchers^1,6,7^ reported that AS was positively correlated, but BD was negatively correlated to SOM. The increased SOM associated with improved soil aggregation enhances soil microbial biomass and activity and N mineralization^1,22^. Soil aggregation has been shown to be positively correlated to C mineralization^23,24^, WEC^25^, and BG activity^11^. Norkaew et al.^4^ reported that WSA was positively related to SOM, STN, PNM, MAC, but BD was negatively related to these parameters. It is likely that soils get less dense, as SOM is increased, favoring soil biological and biochemical properties. The positive associations among IAWHC, WETN, PNM, WEON, CO_2_-1d, NO_3_-N, and PLFA suggest that increased soil water holding capacity can enhance N mineralization and availability by stimulating microbial biomass and activity. Rice and Havlin^26^ reported that soil water content was linearly related to PNM. Similarly, the positive associations among SHC, NO_3_-N, and WEON indicate that increased water infiltration capacity may enhance N availability by increasing N mineralization through increased aeration. In contrast, the negative associations among clay concentration and ACEP, WEON, and BG indicate that increased clay concentration might inhibit N availability by reducing enzyme activity.

#### Relationship between soil physical properties and crop yields

The positive relationships among ASA, WASI, IAWHC, clay concentration, and mean crop yields across years suggest that improved soil aggregation, water holding capacity, and fine-textured soils increased crop yields probably by enhancing soil water retention and availability, aeration, nutrient cycling, and root growth, as soil aggregation promotes these variables^1,22^. Several researchers^3,15^ found that soil aggregation and stability as well as soil water content were positively related to crop yields. Our results are in contrast to those reported by Sene et al.^14^ who observed that soil aggregation was negatively related, but SHC was positively related to crop yield. In contrast, the negative relationship between BD and crop yield indicates that increased soil compaction reduced crop yield, likely due to reduced porosity, aeration, water and nutrient movements, and root growth. This was due to reduced BD from long-term continuous cropping systems which increased annualized crop yields in contrast to increased BD from crop-fallow systems which increased BD but lowered annualized crop yields. Many researchers^15,17,27^ reported that BD was negatively related to crop yield. However, Logsdon and Karlen^2^ demonstrated that BD had no effect on crop yield.

#### Implications of soil physical properties

The ASA, WASI, and IAWHC were associated with most soil physical, chemical, biological, and biochemical properties, followed by clay concentration (Figs. 1, 2, and 3). The BD and SHC were minimally associated with soil properties. The ASA, WASI, and IAWHC were also weakly to very strongly related to mean crop yields across years at Froid and Sidney (Figs. 4 and 5). Clay concentration, BD, and SHC were either not related or weakly to strongly related to crop yield.

These results suggest that ASA, WASI, and IAWHC can be regarded as potential soil health indicators that were associated with most soil physical, chemical, biological, and biochemical properties and related to mean crop yields across years. However, it takes a long time to measure WASI and IAWHC. Soils need to be oven dried for 2–3 days and aggregates need to be separated in a stack of sieves in water to measure WASI. Similarly, soil water contents need to be determined at various hydraulic pressures and soils oven dried for several days to determine IAWHC. In contrast, the determination of ASA is simple, rapid, and inexpensive where the slaking resistance of aggregates is measured by a smart phone loaded with SLAKES software that calculates areas covered by aggregates before and after slaking in water for 10 min. Therefore, ASA can be considered as a potential soil health indicator under dryland cropping systems in arid and semiarid regions.

## Materials and methods

### Experimental details

The long-term experiments occurred at two dryland farming sites (Froid and Sidney) in eastern Montana, USA. The 36-yr-old experiment at Froid (48° 20′ N, 104° 29′ W, elevation 617 m) had a Dooley sandy loam (fine loamy, mixed, frigid, Typic Argiboroll) soil with sand, silt, and clay concentrations of 645, 185, and 170 g kg^−1^, respectively, SOC 14.9 g kg^−1^, and pH 6.2 at the 0–15 cm depth at the beginning of the experiment in 1983. The site had mean annual air temperature (30-yr average) of 8 °C and average annual precipitation of 357 mm. The 14-yr-old experiment at Sidney (48° 33′ N, 104° 50′ W, elevation 592 m) had a Williams loam (fine-loamy, mixed, superactive, frigid, Typic Argiustolls) soil with sand, silt, and clay concentrations of 350, 325, and 325 g kg^−1^, respectively, pH 7.2, and SOC 13.2 g kg^−1^ at the 0–20 cm depth at the initiation of the experiment in 2006. The site had mean annual air temperature of 8 °C and average annual precipitation of 340 mm. The distance between the two sites was 88 km.

Aase and Pikul^27^ described the treatments and crop management for the Froid site and Sainju and Alasinrin^28^ for the Sidney site. In short, treatments at Froid had four cropping systems which were fall and spring till continuous spring wheat (FSTCW), no-till continuous spring wheat (NTCWA), no-till spring wheat-barley (1984–1999) replaced by spring wheat-pea (2000–2019) (NTWPA), and spring till spring wheat-fallow (STWF), each replicated for four times. In each cropping system, each phase of the crop appeared in every year. Tillage in FSTCW occurred with a tandem disc in the fall and a field cultivator in the spring to a depth of 8 cm to prepare the seedbed. Similarly, tillage in STWF occurred with a field cultivator to a depth of 8 cm in the spring and during the fallow period as needed to control weeds. In other treatments, tillage was not applied. The control treatment was STWF which is a conventional cropping in the region. The size of the plot was 12 m × 30 m.

At Sidney, treatments were cropping system as the main plot and N fertilization as the split-plot factor arranged in a randomized block design with three replications. Cropping systems included conventional till barley/spring wheat-fallow (CTWF, traditional system), no-till barley/spring wheat-fallow (NTWF), no-till continuous barley/spring wheat (NTCWB), and no-till barley/spring wheat-pea (NTWPB). In each treatment, barley was grown from 2006 to 2011, which was replaced by spring wheat from 2012 to 2019. All phases of crops in the rotations occurred in each year. Tillage in CTWF was performed with a field cultivator to a depth of 8 cm to prepare seedbeds in the spring and during the fallow period to control weeds. Tillage was not applied to other treatments. Nitrogen fertilizer was broadcast to barley at 0 (N0) and 80 kg N ha^−1^ (N1) from 2006 to 2011 and to spring wheat at 0 (N0) and 100 kg N ha^−1^ (N1) from 2012 to 2019. At both sites, N fertilization rates were adjusted to soil residual N to a depth of 60 cm determined in the autumn of the previous year. Therefore, N fertilization rates included both soil and fertilizer N. Pea did not receive N fertilizer. The size of the split-plot was 12.0 × 6.0 m.

At both sites, spring wheat, barley, and pea were seeded at recommended seed rates with a no-till drill at a row spacing of 20 cm in late April of each year. A banded application of P and K fertilizers at 11 kg P ha^−1^ and 27 kg K ha^−1^, respectively, was done to each crop at seeding. Herbicides and pesticides were applied to each crop as needed. In mid-July to early August, crops were harvested with a combine from a swath of 11.0 × 1.5 m and grain yield was determined after oven drying subsamples at 65 °C for 7 days. After grain harvest, crop residues were returned to the soil.

Soil sampling to a depth of 15 cm and analysis of physical, chemical, biological, and biochemical properties were described in detail by Sainju et al.^29^. The references for methods of analysis of soil properties and abbreviations of parameters are shown in Table 2. For determining BD, two core (3.5 cm inside diameter) samples to a depth of 7.5 cm were collected within a plot and oven dried at 110 °C for 24 h. The BD for a treatment was calculated as the mean values of two cores by dividing the weight of oven-dried soil by the volume of the core^30^. The SHC was determined with an infiltrometer using the two ponding head method after measuring the water flow rate to a depth of 10 cm^31^. Sand, silt, and clay concentrations were determined using the pipette method^32^. The WASI was calculated as the mean-weight diameter of aggregates that passed through 1.00, 0.25, and 0.053 m sieves after shaking the aggregates in water for 10 min^33^. For this, field-moist soil was oven-dried at 55 °C for 2–3 days and passed through a 4.75 mm sieve, out of which 19 g soil was sieved in a stack of sieves in water. The mean-weight diameter of aggregates was calculated by dividing the sum of the products of weight and diameter of each aggregate by the total weight of the aggregates. An image recognition software smartphone app “SLAKES” was used to determine ASA where non-sieved aggregates, about 4–10 mm in diameter, were dispersed in a petri dish and the picture of the area covered by the aggregates was taken by a hanging smartphone above the Petri dish^34^. The areas covered by aggregates before and after immersing aggregates in water for 10 min were calculated by the software and then compared for measuring the slaking resistance of the aggregates. The IAWHC was determined as the difference between volumetric water contents at 33 and 1500 kPa which were determined by using the pressure plate technique^35^.

**Table 2:** Determination of soil physical, chemical, biological, and biochemical properties.

Soil properties	Abbreviations	Reference numbers
**Soil physical properties**		
Bulk density	BD	^30^
Volumetric water content at field-moist condition, water saturation, and at 0.3, 10, 33, and 1500 kPA	VWCFM, VWCFS, VWC0.3, VWC10, VWC33, VWC1500	^37^
Water holding capacity for intact and repacked cores	IAWHC and RAWHC	^35^
Saturated hydraulic conductivity	SHC	^31^
Sand, silt, and clay concentrations	Sand, silt, and clay	^32^
Aggregate stability	AS	^38^
Dry aggregate stability index	DASI	^39^
Water-stable aggregation	WSA	^39^
Wet aggregate stability index	WASI	^33^
Average slake aggregate	ASA	^34^
Stone content	SC	^40^
Macro- and Mesoporosity	MAP and MEP	^41^
Total shrinkage	TS	^40^
**Soil chemical properties**		
pH	pH	^42^
Electrical conductivity	EC	^42^
Buffer pH	BpH	^42^
Al, As, B, Ba, Ca, Cd, Co, Cr, Cu, Fe, K, Mg, Mn, Na, Ni, Pb, S, and Zn concentrations	Al, As, B, Ba, Ca, Cd, Co, Cr, Cu, Fe, K, Mg, Mn, Na, Ni, Pb, S, and Zn	^43^
Inorganic and organic P concentrations	IP and OP	^44^
Sodium-absorption ratio	SAR	^45^
**Soil biological and biochemical properties**		
Soil total C and N	STC and STN	^46^
Soil inorganic C	SIC	^46^
Soil organic C	SOC	^46^
Water-extractable C and N	WEC and WEN	^47^
NH_4_-N and NO_3_-N concentrations	NH_4_-N and NO_3_-N	^44^
KMnO_4_-extrctable C	POXC	^48^
CO_2_ flush at 1-d and 4-d incubations	CO_2_-1d, CO_2_-4d	^49^
Phospholipid fatty acids	PLFA	^50^
Autoclaved citrate-extractable protein	ACEP	^38^
β-glucosidase	BG	^51^
N-acetyl β-glucosaminidase	NAG	^52^
Phosphomonoesterase	PME	^53^
Arylsulfatase	AST	^54^

### Statistical analysis of data

The Pearson’s correlation analysis was used to correlate soil physical properties. To determine the associations among soil physical, chemical, biological, and biochemical properties, a multivariate regression analysis, e.g. PCA using the PROC FACTOR procedure of SAS^36^ was used to analyze the soil data. Data were loaded into two principal components (PC1 and PC2) that explained most variations among physical properties due to treatments. Soil properties were considered positively associated with each other when they are at acute angles, negatively associated when at right angles, and not associated when at obtuse angles. Regression analysis was used to explore the relationships between soil physical properties and mean crop yields across years for individual and combined sites. For these, soil physical properties were used as independent variables and crop yields as dependent variables. Because data for crop yields were absent during the fallow periods in alternate years and also for the latest year and yields can be affected by long-term management, it was decided to use mean crop yield across years for each treatment rather than the yield for the latest year to relate with soil physical properties. Mean crop yield was determined by dividing total yield by the number of years. Yield during the fallow period was considered zero for data analysis. Statistical significance was observed at *P* ≤ 0.05, unless mentioned otherwise.

## Conclusions

The ASA, WASI, IAWHC, BD, clay concentration, and SHC varied in their relationships to soil properties and mean crop yields across years under dryland cropping systems at Froid and Sidney, Montana, USA. The ASA, WASI, and IAWHC were associated with most soil physical, chemical, biological, and biochemical properties and also weakly to strongly related to mean crop yields across years at both sites. The BD, clay concentration, and SHC were less associated with soil properties and varied in their relationships to crop yields. As a result, ASA, WASI, and IAWHC can be used as potential soil health indicators. However, it takes long time to measure WASI and IAWHC. Because of its simplicity, rapidity, and inexpensiveness as well as strong relationships to other soil properties and crop yields, ASA may be considered as an attractive potential soil health indicator under dryland cropping systems in arid and semiarid regions. As soil aggregation is an important parameter that enhances water infiltration, C sequestration, microbial activity, and root growth and decreases soil erosion, using ASA as a physical indicator of soil health not only reduces the cost of soil analysis, but also increases crop yields and environmental sustainability by improving the physical condition of soil, recycling nutrients, reducing soil erosion, and promoting soil C stocks that mitigate climate change.

## Data Availability

The datasets generated during and/or analyzed during the current study are available from the corresponding author on reasonable request.
